# Hypoplastic Left Heart Syndrome Practice Variation Across 31 Centres From 20 European Countries. An AEPC Imaging Working Group Study

**DOI:** 10.1007/s00431-025-06175-9

**Published:** 2025-05-31

**Authors:** Massimiliano Cantinotti, Inga Voges, Giovanni di Salvo, Almudena Ortiz-Garrido, Tara Bharucha, Heynric Grotenhuis, Anna Sabate-Rotes, Anna Cavigelli, Arno Roest, Skaiste Sendzikaite, Oscar Nolan, Tristan Ramcharan, Karel Koubsky, Henrik Brun, Andreas C. Petropoulos, Hannah Bellsham-Revell, Anna Kaneva-Nencheva, Senka Mesihovic Dinarevic, Mohammad Ryan Abumehdi, Gylfi Óskarsson, Peter Olejnik, Gabriela Doros, Tiina Ojala, Thomas Salaets, Jan Sunnegård, Misha Bhat, Julie Wacker, Håkan Wåhlander, Inguna Lubaua, Ulrike Herberg, Owen Miller, Colin J. McMahon

**Affiliations:** 1 Fondazione CNR-Regione Toscana G. Monasterio (FTGM), National Research Institute (CNR), Pisa, Italy; 2 Department for Congenital Cardiology and Pediatric Cardiology, University Hospital Schleswig-Holstein, Campus Kiel, Kiel, Germany; 3 Department Pediatric Cardiology, Padua, Italy; 4 Department of Paediatric Cardiology, Hospital Materno Infantil of Malaga, University of Malaga, Málaga, Spain; 5 Department of Paediatric Cardiology, University Hospital Southampton NHS Foundation Trust, Southampton, England; 6 Department Pediatric Cardiology, Wilhelmina Children’s Hospital/UMCU, Utrecht, The Netherlands; 7 Cardiología Pediátrica, Vall d‘Hebron Hospital Campus, Barcelona, Spain; 8 Cardiology, Children’s Hospital Zurich, Zurich, Switzerland; 9 Department of Pediatrics, Division of Pediatric Cardiology, Willem-Alexander Children’s Hospital, Leiden University Medical Centre, Leiden, Netherlands; 10 Clinic of Paediatrics, Institute of Clinical Medicine, Vilnius University, Vilnius, Lithuania; 11 Department Pediatric Cardiology, Leicester, England; 12 Department Pediatric Cardiology, Birmingham, England; 13 Children’s Heart Centre, Faculty of Medicine, Charles University in Prague and Motol University Hospital, V Úvalu 84, Prague, Czech Republic; 14 Department of Paediatric Cardiology, Oslo University Hospital, Oslo, Norway; 15 “Aziz Aliyev” National Postgraduate Training Center, Baku, Azerbaijan; 16 Evelina Children’s Hospital, London, England UK; 17 Department of Paediatric Cardiology, National Heart Hospital, Sofia, Bulgaria; 18 ANUBIH, Bosnia and Herzegovina, BiH Bosnia, Bosnia and Herzegovina; 19 Department of Paediatric Cardiology, Royal Brompton Hospital, London, England; 20 Department of Paediatric Cardiology, Children’s Hospital Reykjavik, Landspitali University Hospital, Reykjavik, Iceland; 21 Faculty of Medicine, Comenius University, Bratislava, Slovakia; 22 Victor Babes“UMF Timisoara, IIIrd Pediatric Clinic, “Louis Turcanu” Emergency Hospital for Children, Timisoara, Romania; 23 Pediatric Cardiology, University of Helsinki and Helsinki University Hospital, Helsinki, Finland; 24 Department Paediatric Cardiology, University Hospitals Leuven, Leuven, Belgium; 25 Pediatric Heart Centre, The Queen Silvia Children´s Hospital University Hospital, University of Gothenburg, Gothenburg, Sweden; 26 Department of Pediatric Cardiology, Children’s Heart Center, Skåne University Hospital in Lund, 221-85 Lund, SE Sweden; 27 Pediatric Cardiology Unit, Department of Woman, Child and Adolescent Medicine, Children University Hospital of Geneva, Geneva, Switzerland; 28 Riga Stradins University, Clinical University Hospital LV, Riga, Latvia; 29 University Hospital RWTH Aachen, Aachen, Germany; 30 Department Paediatric Cardiology, Children’s Health Ireland at Crumlin, University School of Medicine, University College, Dublin, Ireland

**Keywords:** Hypoplastic left heart syndrome, Practice, Variation, Imaging, Management, Congenital heart disease

## Abstract

Despite significant advances in knowledge and the development of guidelines, the management of hypoplastic left heart syndrome (HLHS) remains highly variable. A structured questionnaire was circulated across European Association of Paediatric & Congenital Cardiology (AEPC) affiliated centres. The aims were to evaluate standards in pre-operative assessment, types of surgery, follow-up and medical practices in children with HLHS. Thirty-one centres from 20 countries completed the survey. Delivery of babies with HLHS occurred in co-located maternity hospitals in 74% of centres; 29% were planned for spontaneous onset of labour, while 54% decided on a case-by-case basis. The preferred initial palliation was a right ventricle-pulmonary artery conduit in 55% of cases, modified Blalock-Thomas Taussig shunt (mBTTS) in 35%, and hybrid in 15% of cases. Timing for Glenn varied from 3 to 6 months of age and preoperative examination varied greatly: 65% performed cardiac catheterization and only 19% performed cardiac magnetic resonance. Stage III palliation was performed at a highly variable interval (2—6 years of age), nearly always employing an extracardiac conduit. Fenestration was routinely performed in 61% and reserved for borderline cases in 39%. All the centers adopted warfarin for the first 3–12 months after Fontan completion, and continued if a fenestration was present, while in non-fenestrated aspirin was left by most centers (e.g. 68%). However, there was a high disparity in the use of heart failure medications (e.g. in interstage I-II 35% use ACE-inhibitors, and only 26% digoxin). Follow-up practice also varied widely with only 60% employing specific protocols. *Conclusion*: This first multi-centre European survey from 31 centres from 20 different European countries highlighted a high practice variation in HLHS management across all the stages of Single Ventricle (Fontan) palliation. Major variations pertained to pre- and post-surgical investigations, surgical strategy for stage I and III, medical treatment regimens, and follow-up programs.

**Table:** 

**What is Known:**
• *Hypoplastic left heart syndrome (HLHS) remains one of the most complex and challenging congenital cardiac defects to manage.*
• *Investigating the management of children with HLHS across different European centres can facilitate study of the most effective management strategies.*
**What is New:**
• *Significant variation in HLHS management were reported in relation to pre- and post-surgical examinations, surgical strategy at stage I and III, medical treatment regimens, and follow-up programs.*
• *Greater standardisation of imaging and diagnostic evaluation, medical treatment and follow-up surveillance may improve outcomes for these vulnerable patients and warrants further study.*

**Supplementary Information:**

The online version contains supplementary material available at 10.1007/s00431-025-06175-9.

## Introduction

In the last few decades, enormous advances have been achieved in our scientific knowledge, clinical practice, and the development of guidelines [1–7] in the management of neonates, infants, and children with hypoplastic left heart syndrome (HLHS). Despite this, the everyday practice of HLHS management through different stages of single ventricle (Fontan) palliation remains highly variable in North America, Australia, and even across European countries, sometimes even showing high practice variation at different centers within the same country [2–5].

Practice variation in HLHS management encompasses differences in prenatal assessment, perinatal care [8, 9], surgical approaches [10–12], and follow-up strategies [2–5]. These variations often stem from institutional experience, resource availability, and differing interpretations of existing evidence, leading to discrepancies in decision-making. The most significant surgical variations occur during Stage I palliation, which includes three main approaches: [1] Norwood with a modified Blalock-Thomas-Taussig shunt (mBTTS), [2] Norwood with Sano modification using a right ventricle-to-pulmonary artery (RV-PA) conduit, and [3] a hybrid approach combining surgical and interventional techniques (pulmonary artery banding with ductal patency maintenance) [10–12].

For Stage III palliation (Fontan completion/total cavo-pulmonary connection, TCPC), variations exist regarding the use of surgical fenestration between the TCPC pathway and the common pulmonary venous atrium [10–13]. The decision to fenestrate is influenced by factors such as patient hemodynamics, center-specific outcomes, and the perceived trade-off between early postoperative stability and long-term complications. Recent meta-analyses suggest minimal differences in physiological and practical outcomes between fenestrated and non-fenestrated Fontan patients, including pulmonary artery pressure, cardiac output, length of stay, mortality, and morbidity [14]. Given these mixed findings, there is no clear consensus on patient selection for fenestration, though it may benefit high-risk patients, such as younger individuals or those with elevated pre-operative pulmonary artery pressures [14]. Similarly, follow-up strategies differ widely across centers, primarily influenced by institutional protocols and resource availability [2–6]. No universally adopted surveillance program for HLHS exists, with significant variability in follow-up visit frequency and the type of investigations performed [5, 15–17].

Preoperative assessment varies based on institutional resources and preferred management strategies, incorporating echocardiography alone, cardiac catheterization, cardiac magnetic resonance imaging (cMRI), or computed tomography (CT) [15–17]. The introduction of advanced imaging techniques, such as MRI lymphangiography and lymphatic interventional procedures, adds further variability, particularly in the long-term management of late complications [18–20].

Pharmacological management remains debated, particularly regarding the use of angiotensin-converting enzyme (ACE) inhibitors and digoxin [21–24]. Additionally, the optimal anticoagulation strategy following TCPC, whether formal anticoagulation (e.g., warfarin, novel oral anticoagulants) or antiplatelet therapy (e.g., aspirin)—remains controversial [25–27].

The aims of this present study are to evaluate current practice across a large number of European centres with regard to the following:

1. pre-operative assessment and management (including prenatal diagnosis, type and setting of delivery
2. imaging examinations performed before different steps of palliation
3. types of surgery
4. follow-up (presence of structured programs, types of examination performed, frequencies of follow-up), and
5. medication strategies (anti-aggregation/anticoagulation policy, heart failure drugs) in neonates, infants, and children with HLHS at various stages of Fontan palliation.

## Methods

A structured and approved detailed survey (Appendix 1) was constructed by the Association for European of Pediatric & Congenital Cardiology (AEPC) imaging working group (IWG) management Committee and circulated to IWG members. All eligible members (seventy) were asked to ensure that a single, unambiguous response, reflective of center policy (and not based on responder's personal view), was provided within the same center to prevent different physicians within the same group from providing inconsistent answers. A retrospective check was also conducted to identify any potential duplicates. Only centers having cardiac surgery were included in the survey. The survey was in Survey monkey® format to facilitate ease of completion. If there was no answer to the initial invitation a second and third email was circulated to the centre delegate. Members were deemed unresponsive/uncontactable after three efforts to engage with the study organisers.

The survey has been divided into 6 majors blocks as follows:

1. Prenatal diagnosis and perinatal/neonatal management,
2. Stage 1 of Fontan Palliation
3. Stage 2 of Fontan Palliation
4. Stage 3 of Fontan Palliation
5. Follow-up after Fontan completion
6. Outcome

The ethics department at Children’s Health Ireland, Crumlin, Dublin, Ireland waived ethical approval for this survey-based study. All participants consented to participate in the study.

### Definitions

‘Imaging Working Group’ is a specialist working group of the Association for European Paediatric and Congenital Cardiology society, which is focused on cardiac imaging, specifically echocardiography, magnetic resonance imaging and computed tomography. The management committee consists of eight elected office holders. ‘Imaging working group members are all Association for European Paediatric and Congenital Cardiology members who register as belonging to the cohort of paediatric cardiologists who are trained, practice and work in cardiovascular imaging. There are approximately 200 IWG members, and this is the largest specialist interest group within the AEPC organization.

## Results

Paediatric cardiologists from 40 AEPC affiliated European cardiology centres were initially invited to participate in the survey. After repeated invitations, delegates from thirty-one paediatric cardiology centres (77% response rate) from 20 different European countries (Azerbaijan, Bosnia Herzegovina, Belgium, Bulgaria, Czech Republic, Finland, Greece, Iceland, Ireland, Italy, Latvia, Lithuania, Netherlands, Norway, Romania, Slovakia, Spain, Sweden, Switzerland, United Kingdom) completed the survey. Activity volumes in the responding centres varied significantly with a median annual number of cardiac surgery cases of 350 (ranging from 0–800) and median number of yearly Norwood operations of 8 (range from 0 to 20) (considering the last 5 years of activity).

## Part 1: Prenatal diagnosis and neonatal management

### Prenatal diagnosis

An antenatal diagnosis rate varied from 50 to 100%, with 16 centers (53%) with an antenatal diagnosis rate > 90%. Counselling to HLHS parents was performed by the fetal medicine team in 19 of the responding centers (60%), and less commonly by a paediatric/fetal cardiologist (6 centers, 20%) or by a palliative care team (6 centers, 20%). Fetal diagnosis with HLHS at a single institution per year varied from 4 to 40 cases per year. The median reported cases of fetal diagnosis of HLHS was 9.25 (interquartile range IQR 5–12) and the median reported cases of live birth of HLHS was 5.5 (IQR 2.5.−7.5), suggesting a 40.5% (IQR 37.5%−50%) attrition rate (termination or spontaneous loss).

Fetuses with HLHS were delivered in a maternity center co-located with a pediatric cardiology unit in 23 of the responding centers (74%) or at a nearby maternity center with postnatal/neonatal transfer to cardiology in 10 centers (31%). A spontaneous onset of labor approach was adopted in 9 of the responding centers (29%), induction of labor in 4 centers (12%), elective Caesarean section in 2 centers (6%), while in most of the responding centers (16, 53%) did not have a uniform strategy.

### Neonatal management and examination before Norwood operation

**Fig. 1:**
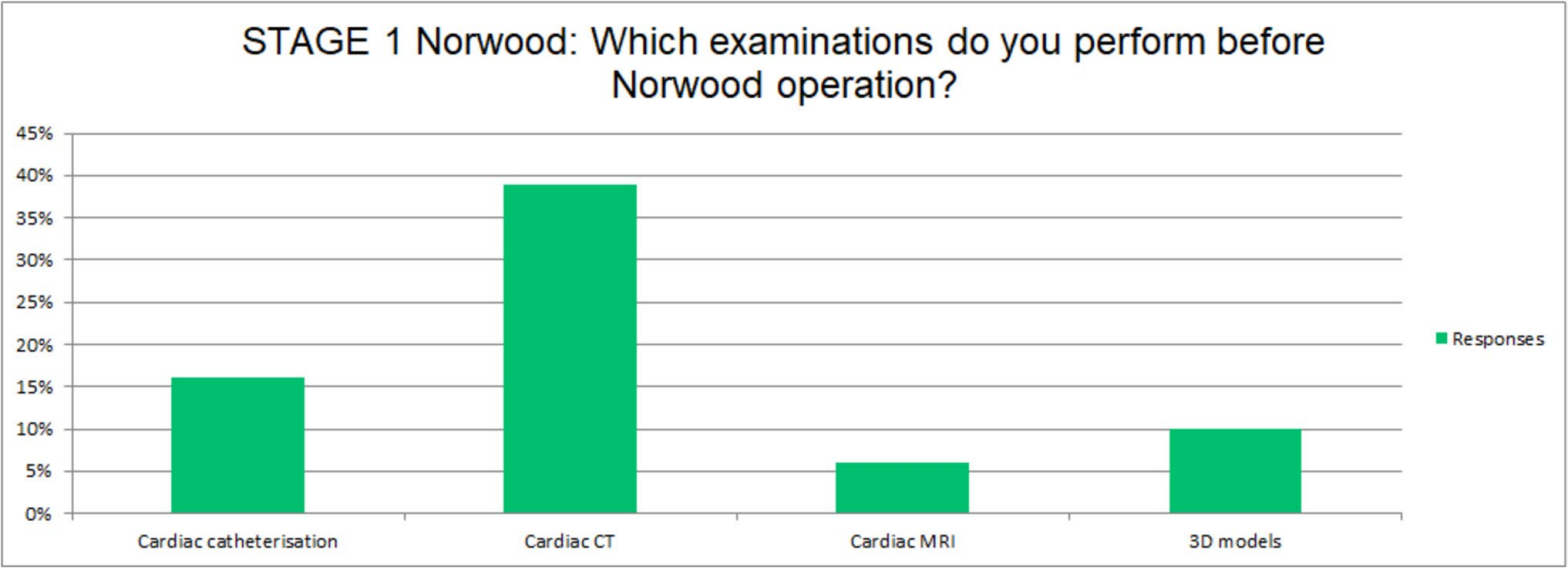
Examination performed before Norwood operation at different centres

Twenty-three (75%) of the responding centers admit neonates with HLHS to an intensive care unit, while 8 (25%) initially manage these patients on a general pediatric ward. Echocardiography is a universal imaging modality in the diagnosis of HLHS, but many other cardiac imaging modalities are employed in the preoperative assessment before Norwood Stage 1 surgery, including cardiac CT (12, 39%), cardiac catheterization (5, 16%), and cMRI (2, 6%). Three-dimensional (3D) models are used to assist surgical planning for Stage I palliation in only 3 centers (10%). (Fig. 1).

## Part 1: Stage 1 of Fontan Palliation

### Preferred initial palliation

**Fig. 2:**
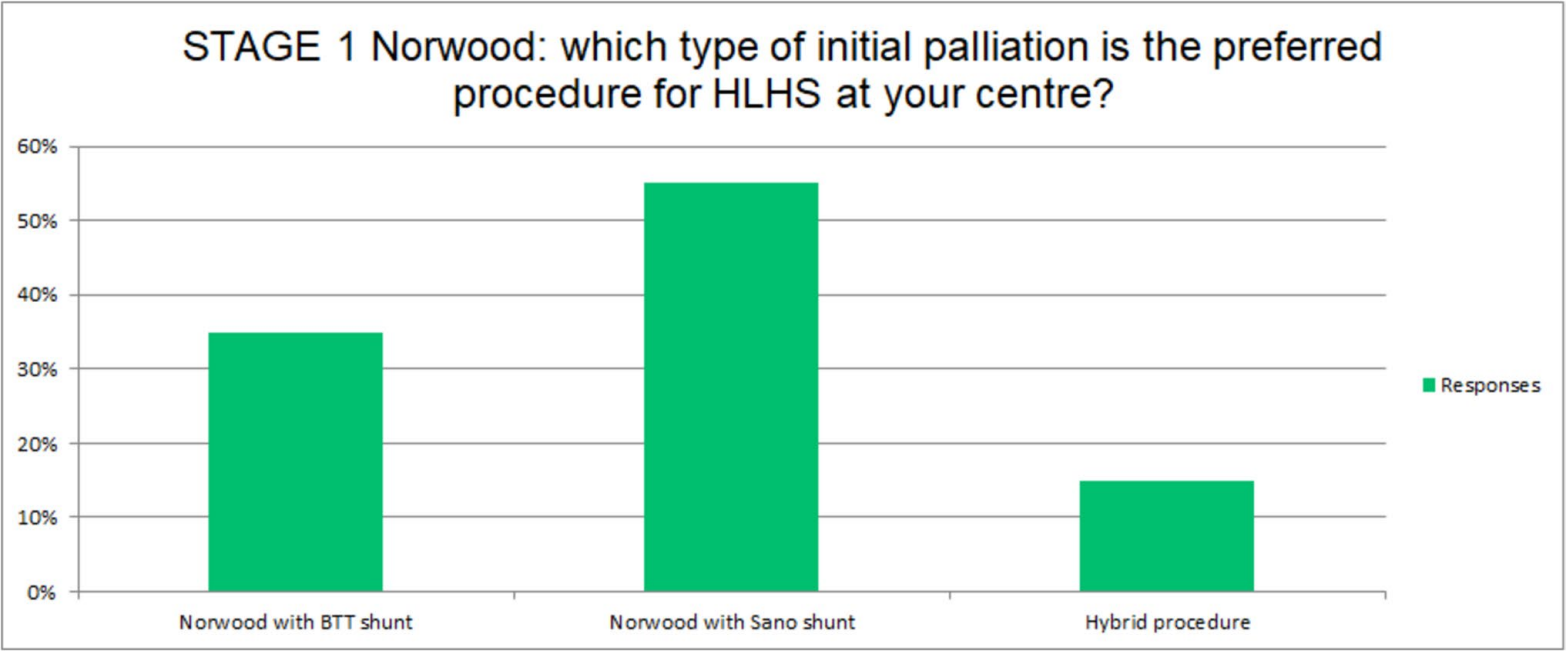
Preferred initial palliation procedure at different centres

In 17 (55%) of the responding centers, the preferred initial palliation for HLHS is Norwood with Sano (RV-PA conduit) modification, in 11 (35%) a classical Norwood with mBTTS is employed, while in the remaining 6 (20%), the hybrid procedure is the preferred approach (Fig. 2). Overall, hybrid procedures are available and performed at least selectively in 9 (30%) of the participating centers, with equal numbers using prostaglandin to maintain ductal patency or ductal stenting. Among centers performing the hybrid intervention, standardized indications for this procedure (including prematurity, low birth weight, severely hypoplastic ascending aorta, right ventricular dysfunction, severe tricuspid regurgitation, comorbidities, and genetic disorders), are present in 13 (41%), while in the remaining there are not uniform indications for hybrid intervention.

### Postoperative Follow-up and Management After Discharge from Stage 1

**Fig. 3:**
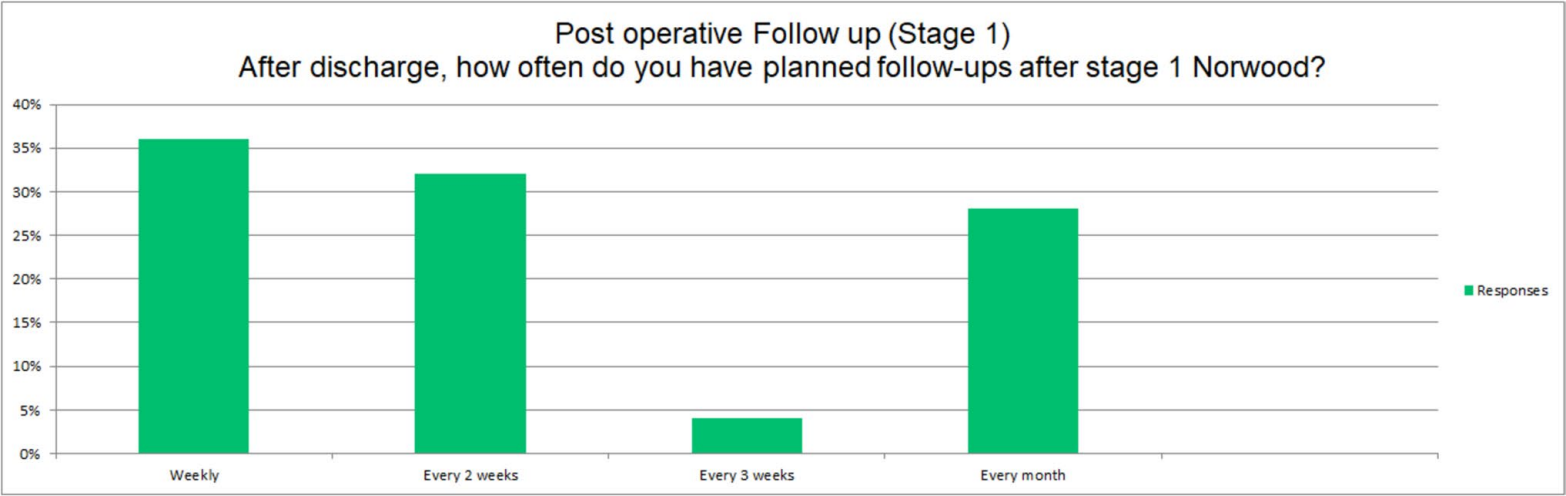
Follow-up interval after Stage 1 Norwood palliation at different centres

Eighteen (58%) of the responding centers discharge HLHS patients from the hospital during the interstage period (between Stages I-II), 1 (3%) retain patients in the hospital until Stage II (Bidirectional Glenn), while 12 (39%) discharge selectively depending on patient factors. If discharged after Stage I, follow-up intervals vary: Weekly (11 centers, 35%), every 2 weeks: (10 centers, 32%), every 3 weeks (2 centers, 6%), monthly (7 centers, 23%) (Fig. 3).

During inter-stage follow-up, all responding centers assess body weight and pulse oximetry (SpO2). In 30 centers (97%), echocardiography is performed; in 25 (80%), an electrocardiogram (ECG) is performed; in 2 (6%), a 24-h ambulatory ECG is conducted; and in 2 (6%), a cMRI is undertaken. Home Monitoring Programs (HMPs) with weekly SpO2 and body weight assessments are present in 15 (48%) of the responding centers. Monitoring with SpO2 alone is available in 2 (6%), and weight monitoring alone is used in 1 (3%) of centers (Fig. 1). An individualized program is present in 5 (16%), and telemedicine is utilized in 2 (6%) of the responding centers. 8 (27%) of the respondents do not use any form of home monitoring.

### Medication Employed in the Interstage Period (Between Stages I-II)

Aspirin is used by 28 (90%) of the responding centers, and clopidogrel is used by 5 (16%). Other medications employed include diuretics (17 centers, 55%), digoxin: (8 centers, 26%), ACE inhibitors (11 centers,35%), and beta-blockers (4 centers,13%) (Fig. 2).

## Part 3: Stage 2 of Fontan Palliation

### Surgical Timing, Pre-operative Assessment, and Follow-up for Superior Cavo-Pulmonary Anastomosis (Bidirectional Glenn or Hemi-Fontan)

The planned surgical timing for Stage II superior cavo-pulmonary anastomosis (Bidirectional Glenn or Hemi-Fontan) is 4–6 months in 14 (45%) of the responding centres, 6 months in 9 (30%), and 3–5 months in 8 (25%).

**Fig. 4:**
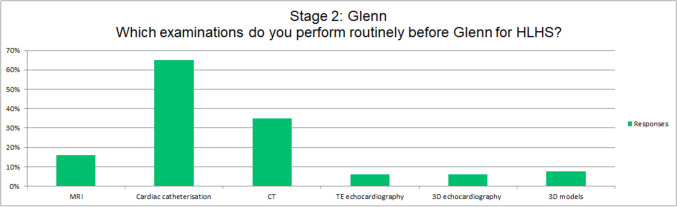
Pre-operative examination before Glenn at different centres

Preoperative assessment before Stage II superior cavo-pulmonary anastomosis for HLHS varies significantly across centres. Cardiac catheterization is routinely performed in 20 of the responding centres (65%), cardiac CT in 11 centres (35%), cMRI in 5 centres (16%), transesophageal echocardiography in 2 centres (6%), 3D echocardiography for valve morphology in 2 centres (6%), and 3D models in 2 centres (6%). (Fig. 4).

A standardized ambulatory follow-up program is present in 16 (51%) of the responding centres, while in 15 (49%), a structured follow-up program is not available.

## Part 4: Stage 3 of Fontan Palliation

### Pre-operative Assessment in Borderline Conditions for Total Cavo-Pulmonary Connection (TCPC) – Fontan Completion

The ideal timing of Fontan completion varies greatly among centres, from 2 to 6 years of age. Weight preferences also vary from 12 to 20 kg, with 7 (22%) of the responding centres indicating 15 kg as the ideal minimum weight for TCPC.

**Fig. 5:**
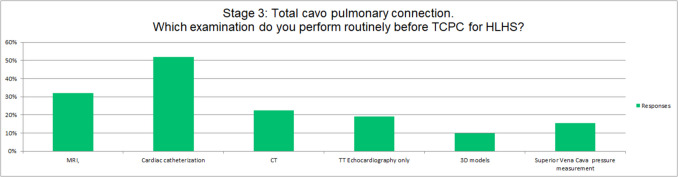
Pre-operative examination before total cavo-pulmonary connection (TCPC) at different centres

Preoperative assessment before TCPC varies significantly among centres: 6 of the responding centres (19%) use echocardiography only, 16 (52%) routinely perform cardiac catheterization, 10 (32%) cMRI, 7 (23%) CT, 3 (10%) 3D models, and 1 (4%) superior vena cava (SVC) cannulation and SVC pressure measurement. (Fig. 5).

Only 5 (17%) of the responding centres attempted to complete Fontan even in high-risk patients with unsuitable parameters, such as elevated pulmonary vascular resistance (PVR), elevated pulmonary arterial (PA) pressure, or significant collateral vessels. When the mean PA pressure was 16 mmHg, 14 of the responding centres (45%) treated with pulmonary vasodilators before reassessment, 8 (26%) completed the Fontan, and 7 (22%) did not have a uniform strategy and made decisions on a case-by-case basis. When mean PA pressure was 18 mmHg, 15 (48%) treated with pulmonary vasodilators and re-evaluated the patient, while 6 (20%) declined Fontan completion, and 10 (32%) did not have a uniform strategy. When the mean PA pressure was 20 mmHg, 16 (52%) treated with pulmonary vasodilators before reassessment, while 7 (23%) declined Fontan completion, and 8 (25%) did not have a uniform strategy.

For patients with elevated PVR, 28 (90%) of responding centres used pulmonary vasodilators (Sildenafil, Bosentan, or other agents) to reduce elevated PA pressure/PVR and improve suitability for Fontan, while 3 (10%) did not attempt this strategy. When a pulmonary vasodilator was used, 15 (48%) re-evaluated the patient after 3–6 months, 7 (23%) after 6 months, 1 (3%) after 6–9 months, 1 (3%) after 6–12 months, while 7 (23%) did not have a uniform protocol, and re-evaluated at different times depending on the case.

### Criteria for Declining Fontan Completion

Factors cited to decline Fontan completion included elevated PVR (26 centres, 85%), elevated PA pressure (26 centres, 85%), small PA size or stenosis (24 centres, 77%), left ventricle/right ventricle end-diastolic pressure (17 centres, 54%), multiple collaterals (14 centres, 46%), ventricular systolic dysfunction (25 centres, 81%), and ventricular diastolic dysfunction (18 centres, 58%).

### TCPC Surgery and Fenestration Policy

Twenty-six of the responding centres (85%) perform an extracardiac TCPC, while the remaining 5 (15%) prefer a lateral tunnel technique. Regarding fenestration, 19 of the responding centres (61%) created a Fontan fenestration for all cases of HLHS, while in 12 centres (39%) there was not a uniform strategy, and the approach was tailored to the individual case. Fenestration size varied from 3 to 6 mm, with the most common size being 4 mm in 11 centres (35%).

If fenestration persisted over time, 15 (48%) of the centres did not close it, 15 (48%) closed it depending on PA pressure, while 1 (4%) routinely closed all fenestrations. Fontan fenestration closure was accomplished at highly variable time intervals, ranging from 3 months to 2 years after Fontan.

When closing the fenestration, 12 of the responding centres (40%) performed balloon occlusion to assess PA stability, 3 (10%) did not, while 16 centres (50%) did not have a uniform strategy on balloon occlusion. All respondents left the fenestration patent when PA pressure was borderline (elevated).

## Part 5: Follow-up after Fontan

### Follow-up Programs

Twenty (66%) of the responding centres have a detailed follow-up protocol after Fontan completion. A dedicated single ventricle clinic, however, was present in only 5 centres. Fontan follow-up was led by a dedicated team of 2–4 cardiologists in 10 (31%) of the responding centres, while in most cases, this duty was shared among a greater number of cardiologists. The majority (respondents from 20 centres, 65%) believed that a dedicated pool of 2–4 cardiologists was preferable for the follow-up of these vulnerable patients. The follow-up interval for the stable Fontan varied greatly among centres, from 4 to 24 months, with the largest group, 9 centres (30%), offering yearly follow-up.

### Medications During Follow-up

Medication strategies after Fontan completion varied significantly. Warfarin was used routinely in the first 3–12 months after surgery and continued if a fenestration was present. In non-fenestrated TCPC patients or when the fenestration was closed, most centres (21, 68%) converted to aspirin therapy after the initial post-surgical period. However, in 10 (32%) of centres, warfarin was maintained indefinitely; Rivaroxaban was used in 8 centres (26%). ACE inhibitors were used in 13 (42%), diuretics in 14 (45%), mineralocorticoid inhibitors in 5 (16%), and cardiac glycosides in 4 (13%) centres.

### Investigations During Follow-up

Yearly blood tests were performed in 25 (81%) of the responding centres, including liver function tests in 28 (92%) and N-terminal pro-brain natriuretic peptide (NT-ProBNP) in 27 (87%). Cardiopulmonary exercise testing was performed in 24 (77%) of centres every 1–2 years, abdominal ultrasound every 1–2 years in 8 (25%), and cMRI every 2–5 years in 21 (69%).

Specific liver ultrasound was performed in 27 (86%). Liver biopsy was performed routinely in 2 (8%) of the responding centres and in selected cases in 10 (31%). Magnetic resonance lymphangiography was performed in 18 (57%), and lymphatic interventional procedures in 13 (43%).

## Part 6: Outcomes

Survival to adulthood (e.g. 18 years of age) varied among centres, ranging from 40 to 100% with a mean survival rate of 58.7% (IQR 50%−70%). A transplantation program for Fontan patients was available at 13 (42%) of the responding centres, at other national centres at 11 (35%), while 7 (23%) required sending patients abroad.

## Discussion

We have performed this first cross-national multi-centre survey on HLHS clinical practice across 31 European centres, involving 20 countries with highly different population, workload, infrastructure, and economical resources. Our study highlighted broad variation in practice but also some similarities and important advances in the management of neonates, infants, and children with HLHS through all stages of the Fontan pathway. Differences emerged across the whole pathway, from fetal programs to postnatal management, in interventional strategies and for the various phases of follow-up. Due to the remarkable advances over the last two decades [8, 9], diagnosis of HLHS was usually performed prenatally in most centres. Our survey highlights a growing tendency toward pregnancy termination, with an attrition rate of 40.5% following a prenatal diagnosis of HLHS (including both termination and spontaneous loss). While our data allow for an exact calculation of pregnancy termination rates after a fetal HLHS diagnosis, it is reasonable to infer that the rate falls between the 9% reported in recent US studies [28] and the 60% observed in some Australian registries [28]. It’s difficult to explain such wide differences among different geographic areas. The decision-making process following an HLHS diagnosis is complex and influenced by multiple factors, including parental perspectives, socio-cultural norms, and healthcare system structures. Soszyn et al. [28] examined factors influencing care pathways in New Zealand and found that geographic location, ethnic background, and socioeconomic status significantly impacted parental decisions.

Disparities among centres emerged regarding the co-location of maternity units and the preferred method of delivery. Co-location of a maternity unit represented the optimal setting for delivery of babies with critical CHD [1, 28, 29]. This was present in 74% of the respondents. Despite evidence supporting the benefits of spontaneous delivery [8, 9], this was the preferred option in only 23% of centres, while 54% did not have a uniform strategy on the mode of delivery.

In accordance with previous observation [3, 4] our survey revealed heterogeneity in the preferred interventional strategy for the first stage of Fontan palliation. Despite recent data not proving superiority of (Sano) modified Norwood with RV-PA conduit [17, 11], this remained the preferred option in 55% of the responding centers, while in thirty-five 35% the mBTTS shunt is preferred. In accordance with recent guidelines [1], in centres preferring mBTTS shunt, the Sano RV-PA conduit was reserved for higher-risk cases. Hybrid procedures [1, 11] instead were performed in only 30% of the responding centers.

Due to the known high risk of interstage mortality [1, 2, 7], close interstage surveillance after Stage-I, was performed universally. However, the surveillance format (complete examination versus O2 saturation and weight monitoring) and the frequency (weekly, biweekly, monthly), varied widely among centres. Unfortunately, 27% of the centres did not have dedicated follow-up programs for Interstage I-II. Most centres planned to discharge infants between Stage I and II, but the decision to discharge was predicated on clinical conditions in almost half of the responding centres.

Timing for bidirectional Glenn varied from 3 to 6 months of age [1], and examinations performed preoperatively varied greatly. Cardiac catheterization was performed in up to 65% of the responding centres before Glenn, and in 52% before TCPC. Whilst a broad literature supporting its use [1, 14, 15], cMRI was employed in only 16% of the centres before Glenn and in 32% before TCPC. In contrast, CT was performed more than MRI (35%) prior to bidirectional Glenn, while its use decreased prior to TCPC (32%). Of interest, 19% of centres used echocardiography only prior to Fontan completion/TCPC.

3D echocardiography, which may be helpful for the assessment of tricuspid valve morphology [30], was infrequently employed. Similarly, 3D models, which may add additional pre-surgical information [31, 32], were rarely employed.

Quite surprisingly there was little agreement on the ideal timing for Fontan completion, with a great variation among centres from 2 to 6 years of age. Stage III was almost universally accomplished using an extracardiac TCPC with decisions pertaining fenestration remaining controversial, with more than half of centres creating a fenestration routinely. There were no established criteria to decide suitability for closure of a Fontan fenestration [34], which may explain the variability in practice reported. Our data indicated that 48% do not close Fontan fenestration, 48% tend to close depending on PA pressure, while a minority (4%) reported that they close the fenestration routinely.

HLHS with elevated pulmonary arterial pressure is another subject of contention, with limited evidence [35, 36] on the utility of vasodilators. Criteria to decline TCPC completion varied [6], and strategies to be adopted in cases of borderline PA pressure (from 16 to 20 mmHg) also varied, with most centres treated with pulmonary vasodilators and re-studied patients at a later date.

Regarding antiaggregating/anticoagulation therapy, and in line with current evidence [25, 27], most of centers (e.g. 68%) use of aspirin in low-risk Fontan and warfarin was adopted in all centers for the first 3–12 months after completion of TCPC and continued if a fenestration was present. Aspirin was used by 90% of responders for the first interstage I-II period, while 19% of the responders used either aspirin or clopidogrel during this phase. Aspirin was also common between Glenn and Fontan (interstage II-III) and by the recent availability of non-vitamin K antagonist oral anticoagulants (NOAC) raises the possibility of an easier and more child-friendly anticoagulation medication in Fontan patients [25, 26]. The routine use of NOACs has already been introduced, with 26% of the responders reporting the use of Rivaroxaban.

In accordance with previous observation [21], this survey confirmed a marked heterogeneity in the use of heart failure medications. Previous studies have questioned how, despite widespread use, ACE-inhibitors [22, 31] have no proven evidence of benefit to Fontan patients. ACE inhibitor therapy also did not provide a beneficial effect on interstage failure among infants with HLHS [24]. Conversely the use of digoxin [23], has been reported to show a significant benefit in ameliorating transplant-free interstage survival after the stage I procedure. Ironically digoxin was routinely employed by 26% of centres while ACE-inhibitors continued to be used in 35% of the centers (35%). ACE inhibitors were also employed in 42% of the centres after Fontan completion.

Fontan follow-up is particularly challenging, with an increasing number of investigations available to evaluate cardiac, pulmonary and systemic components of the TCPC circulation [1–6]. Despite 60% of the responding centres having a follow-up program post Fontan, a dedicated pool of cardiologists managed these patients in only 31% of the centres, and the number of specific single ventricular clinics was still limited. Increasing attention was given to liver ultrasound [6, 7], which was performed in 86% of the responding centers, with 8% also performing liver biopsy in selected cases. In the last years there has been greater attention directed to the study of lymphatic function in Fontan patients [18–20]. Despite this examination being relatively new, more responding centres are performing MRI lymphangiography.

The Fontan pathway also poses economical and ethical considerations. Not surprisingly our survey confirmed differences [3–5] in the outcome of HLHS among centres with widely different workload and facilities, including the availability of a transplant service (40% of the responding centres).

## Strengths and Limitations

One of the strengths of this survey is its ability to capture the beliefs and clinical practices of individual cardiologists, which are often key drivers in shaping medical decision-making. Despite potential limitations in the data, it offers valuable insight into how clinical practice is influenced by professional convictions. Additionally, the survey highlights areas where persistent suboptimal or highly variable practices exist, pointing to potential opportunities for quality improvement and further research. However, the authors could further elaborate on the limitations of this survey, particularly regarding the fact that the responses represent proxy markers of centre practices, often reflecting the perspective of a single cardiologist rather than a comprehensive institutional practice. This introduces the possibility of misinterpretation or bias in how centre-wide practices are reported. Some questions in the survey may also have been subject to ambiguity, potentially leading to misinterpretation by respondents. Moreover, the outcomes and survival data derived from this survey should be interpreted with caution, as they are based on self-reported practices rather than patient-derived datasets, which would offer more robust and clinically relevant insights. Also, for some specific points, such as lymphatic procedures questions were quite generic, not requiring data from specific patient-derived dataset.

While previous works have focused on specific aspects of the Fontan pathway this survey offers a comprehensive assessment of clinical practice through all the phases of single ventricle palliation in children with HLHS. Although this is not a complete dataset of all European centres, our survey of 31 centres from 20 countries represents a large and potentially representative sample to study practice variation across a large continent. We did not distinguish between HLHS subtype and anatomical variants [1]. Given the wide spectrum of care involved in HLHS management across the various stages of Fontan palliation, one survey cannot encompass all aspects of care of patients with HLHS. Some important areas such as a comprehensive list of medications at different stages, sports recommendations, and other considerations do warrant further investigation. The aim of the present study however was to provide a broad overview of current practice in major aspects of care. Further studies may be helpful to define variations in specific aspects of the management of HLHS. Recommendations to standardise the care pathway for infants and children with HLHS during the various stages of Fontan palliation) would potentially optimise management of this vulnerable group of patients.

## Conclusion

Despite advances in knowledge and sharing evidence, there is still a great heterogeneity in clinical practice through all stages of Fontan palliation in infants and children with HLHS. Major differences pertain to the investigations performed before and after each stage, surgical strategy (RV-PA conduit versus MBTTS, lateral tunnel versus extracardiac TCPC, to fenestrate or not) and in medications prescribed. Significant variation also remains in the organization of various phases of the univentricular programs and in the cardiac imaging performed at different stages. To identify the most effective surgical, interventional, and medication strategies, multicenter studies with large sample sizes are essential. Additionally, the development of consensus documents or recommendations could play a crucial role in standardizing imaging protocols and follow-up care across different centers. Our survey, however, also reveals a good level of European infrastructure for single ventricular programs (from fetal diagnosis to late follow-up of Fontan) and a willingness to introduce new imaging techniques, treatment procedures and medications, which with greater standardization may translate to better outcomes for these potentially vulnerable patients.

## Supplementary Information

Below is the link to the electronic supplementary material.

ESM 1 (DOCX 25.0 KB)

## Data Availability

Data is available upon request.
