# Supplementary material for: Hypoplastic Left Heart Syndrome Practice Variation Across 31 Centres From 20 European Countries. An AEPC Imaging Working Group Study
Source: Eur J Pediatr. 2025 May 31;184(6):379. doi: 10.1007/s00431-025-06175-9 (PMC12126313; doi:10.1007/s00431-025-06175-9)
Supplement: Supplementary file 1 — (DOCX 25.0 KB) [file 431_2025_6175_MOESM1_ESM.docx]

**Survey Proposal**

**Practice Variation in HLHS and HRH Management:**

**A Pan European Survey Study**

**SURVEY – Practice Variation HLHS patients**

Nation:

Centre:

Center Total Number of Cardiac Surgeries per year:

Total Number of Cardiopulmonary Bypass (CPB) Surgical Cases per year:

Total Number of Non-CPB Surgical Cases per year:

**HLHS**

Number (average) of Norwood operations per year:

Number of Glenn operations for HLHS per year:

Number of TCPC for HLHS per year:

**HLHS Patients**

1. Which type of palliation is the preferred procedure for HLHS at your centre? (respond with one +, or++):
   1. Norwood
   2. Norwood-Sano shunt
   3. Hybrid procedure
      - PA banding with prostaglandin
      - PA banding with ductal stenting

Is there one procedure which is the preferable surgical technique for HLHS?

If you perform both Norwood/BTT shunt and Norwood/Sano shunt at your centre what determines which procedure is chosen?

Indications for Hybrid procedure?

1. Which examination do you perform before Norwood operation (express one or more preferences, one +, or++)?
   1. Echocardiography only
   2. Cardiac catheterization
   3. CT
   4. MRI
   5. 3D models
   6. Others – please elaborate ________________
2. How often do you have planned follow-ups and how detailed (physical examination, echo, electrocardiogram-ECG-, more advanced techniques) after Norwood palliation?
3. Do you have a Home Monitoring Program after the Norwood procedure?
   1. Weekly O2 saturation and weight monitoring?
   2. Weekly O2 saturation monitoring?
   3. Weekly weight monitoring?
   4. Individually tailored by need?
4. Do you discharge HLHS patients between Norwood operation and Glenn operation / Hemi-Fontan operation?
5. Which types of medication do you mostly use after the Norwood procedure?
   1. Aspirin
   2. Beta-blocker (specify the type)
   3. Ace-inhibitors
   4. Cardiac glycoside (Digoxin)
   5. None
   6. Other, specify
6. Ideal age (weight) for Glenn operation for HLHS patients?
7. Ideal age (or weight) for TCPC operation for HLHS patients?
8. Which examination do you perform routinely before Glenn operation for HLHS (express one or more preferences, one +, or++)?
   1. MRI
   2. Cardiac catheterization
   3. CT
   4. Transthoracic Echocardiography only
   5. Trans-oesophageal echocardiography
   6. 3D echocardiography of AV valve function
   7. 3D models
   8. Others
9. Do you have an ambulatory clinic with univocal follow-up protocol for patients after Glenn or Hemi-Fontan operation for HLHS, or are the upcoming follow-up visits decided at discretion of the physician
   1. Follow-up protocol with detailed examinations (physical examination, echo, ECG, more advanced techniques)
   2. No settled follow-up protocol or detailed list for examinations.
   3. On average, what is the time period between the ambulatory visits
10. Regarding the postoperative Glenn phase for HLHS please express one or more preferences, as one +, or++
    1. Aspirin
    2. Beta-blocker (specify the type)
    3. Cardiac glycoside (Digoxin)
    4. ACE inhibitors
    5. ARB inhibitors
    6. Diuretics
       1. Frusemide
       2. Aldactone/spironolactone
    7. -none
    8. Warfarin, period
11. Which examination do you perform routinely before TCPC for HLHS (express one or more preferences, one +, or++)?
    1. MRI,
    2. Cardiac catheterization
    3. CT
    4. echocardiography only,
    5. 3D models,
    6. Superior Vena Cava cannulation and pressure measurement,
       1. others
12. Is MR lymphangiography routinely performed prior to TCPC?
13. What Strategy is used for TCPC
    1. Lateral tunnel with or without fenestration
    2. Extracardiac fenestrated
    3. Extracardiac not fenestrated
    4. Extracardiac fenestrated only in difficult cases
    5. Others, expressed
14. If selective fenestration in some patients, how do you decide who to fenestrate?
15. What size fenestration does your surgeon typically leave?
16. Do you close all fenestrations if they persist over time?
17. Yes
18. No
19. Depends on physiology (PA pressure)
20. How long after Fontan do you occlude the fenestration? (months)
21. Do you balloon occlude fenestration to assess PA pressure is stable?
22. Yes
23. No
24. Depending on the case
25. Will you leave fenestration open in borderline PA pressure cases?
26. Yes
27. No
28. Do you have an ambulatory clinic with univocal follow-up protocol for patients after Fontan completion, or are the upcoming follow-up visits decided at discretion of the physician
    1. Follow-up protocol with detailed examinations (physical examination, echo, ECG, more advanced techniques)
    2. No settled follow-up protocol or detailed list for examination
    3. On average, what is the time period between the ambulatory visits of the stable patient
29. What strategy of antiaggregating/anticoagulation do you prefer?
30. Warfarin for fixed period
31. Warfarin until fenestration closed
32. Aspirin only
33. Aspirin after warfarin ceased
34. Tinzaparin
35. Other anticoagulants
36. Do you change from warfarin to aspirin after 6 months after TCPC?
    1. Yes
    2. No
    3. Only if fenestration closed
37. What examination and how often (indicate months) do you perform follow-up investigations in non-complicated cases? (Express one or more preferences, one +, or++)
    1. MRI
    2. CP stress test
    3. Ultrasound abdomen
    4. Blood tests
    5. X-Ray
    6. Others
38. How often and what type of blood test do you perform? (Express one or more preferences, one +, or++):
    1. NTproBNP/ BNP
    2. Liver enzymes
39. Do you perform liver ultrasound or elastography?
40. Yes
41. No
42. If yes in which cases and how often?
43. From what age do you start liver surveillance?
44. Do you perform Liver biopsy in Fontan patients?
45. Yes
46. No
47. If yes in which cases
48. Do you perform MR lymphangiography at your center?
49. Yes
50. No
51. If yes in which cases
52. Do you perform lymphatic interventional procedures for chylothorax, PLE or plastic bronchitis?
53. Yes
54. No
55. If yes in which cases
56. If no, where do you refer patients to for these procedures?
57. Who perform lymphatic interventional procedures?
    1. Interventional radiologist
    2. Interventional cardiologist
    3. Both radiologist and cardiologist
    4. Other personnel
58. Which criteria do you use to decline Fontan palliation?
    1. PV resistance
    2. PA pressure
    3. PA size and stenoses
    4. LV/RVEDP
    5. Multiple collaterals
    6. Systolic ventricular dysfunction
    7. Diastolic ventricular dysfunction
59. Do you use pulmonary vasodilators (Sildenafil/Bosentan, other agents) to try to reduce PVR and PA pressure in patients with elevated PA pressure/PVR to make them suitable for Fontan?
60. Yes
61. No
62. If you use vasodilators how long do you treat before repeat catheterization of the patient to see if the hemodynamics are favorable?
63. Do you try to complete Fontan at all costs even if patient has some unsuitable parameters (PVR, PA pressure, collaterals)?

Please comment____________________________________________________________

1. As part of pre Fontan assessment if mean PA pressure is 16mmHg what do you do?
   1. Fontan
   2. Decline Fontan
   3. Treat with pulmonary vasodilators and repeat measurements

If mean PA pressure is 18mmHg what do you do?

1. Fontan?
2. Decline Fontan
3. Treat with pulmonary vasodilators and repeat measurements

If mean PA pressure is 20mmHg what do you do?

1. Fontan
2. Decline Fontan
3. Treat with pulmonary vasodilators and repeat measurements
4. Is there a dedicated single ventricle clinic (“survivorship program” in U.S.) in your centre?
5. Yes
6. No
7. If so, is this clinic single cardiology input or multidisciplinary team (cardiologist, electrophysiologist, dietician, hepatologist, psychology, social work)?
8. Single cardiology
9. Multidisciplinary
10. Do 1-2 doctors look after all single ventricles patients or is this shared between all cardiologists at your institution?
11. 1-2 doctors
12. Shared
13. Do you think 1-2 doctors should only look after single ventricle patients or should this be shared between different cardiologists?
14. 1-2 doctors
15. Shared
16. Are single ventricle patients delivered by caesarean section, induction or SVD?
17. Caesarean section
18. Induction
19. Spontaneous delivery
20. Are single ventricle patients scheduled for delivery on a Monday or any day of the week?
21. Are single ventricle patients counselled antenatally by a cardiologist alone or high-risk foetal medicine team (including cardiologist) at diagnosis?
22. What is survival to adulthood (18 years) quoted by your centre for HLHS?
    - - %
23. What is survival to adulthood (18 years) quoted to adulthood for single ventricle hypoplastic right heart syndrome (e.g. tricuspid atresia) at your centre?
    - - %
24. Where is transplantation available for Fontan patients?
25. At you centre
26. Abroad in shared care transplant pathway
27. Do limited resources in your country impact your ability to develop a single ventricle program (MDT multidisciplinary team clinic etc.)?
28. Are babies with single ventricle admitted to the following after birth?
29. Paediatric intensive care unit
30. Neonatal intensive care
31. High dependency unit
32. General paediatric ward
33. Where is maternal hospital co-located:
34. with paediatric hospital
35. at a separate hospital
36. How far away is maternity hospital from paediatric hospital?
37. Given the increasing evidence of the poor fate of systemic single RV patients do you think it is still reasonable to undertake single ventricle palliation in this group (HLHS)?
38. Yes
39. No
40. We have mixed feelings on HLHS long term
41. How many fetuses with HLHS are diagnosed per year at our institution?
42. How many babies with HLHS are delivered per year at your institution?
43. Is there fetal cardiac intervention at your centre for HLHS or critical AS/EFE?
    1. Fetal aortic valvuloplasty
    2. Fetal atrial septal defect creation / enlargement
44. Do you recommend Fontan patients participate in an active exercise program?
45. How long and how frequent do you recommend they exercise per week?
